# Depression, anxiety, and stress impair sperm quality via dysregulation of the mitochondrial PDK–PDC axis

**DOI:** 10.1186/s12958-025-01458-0

**Published:** 2025-09-30

**Authors:** Wang Wang, Wang Qikai, Wang Zilin, Shao Junyan, Jiang Xiaocui, Liu Qi, Chen Shuhui, Zhu Yangyang, Gao Mengjie, Chen Siyi, Cao Jigang, Xiao Min

**Affiliations:** 1 Hubei University of Traditional Chinese Medicine, Wuhan, 430065 China; 2 Wuhan Orthopaedic Hospital of Integrated Chinese and Western Medicine, Wuhan, 430070 China; 3 Hubei Provincial Hospital of Traditional Chinese Medicine, Wuhan, 430061 China; 4 Tongji Medical College, Huazhong University of Science and Technology, Wuhan, 430030 China; 5 Hubei Provincial Integrated Chinese and Western Medicine Hospital, Wuhan, 430015 China

**Keywords:** Male infertility, Sperm motility, Psychological stress, Mitochondrial metabolism, PDK-PDC axis

## Abstract

Male infertility, a significant global public health challenge, arises from multifactorial interactions involving genetic, metabolic, and psychological factors. While psychological stress (depression/anxiety/stress, DAS) is increasingly linked to impaired sperm quality, its molecular mechanisms remain unclear. This cross-sectional study investigates mitochondrial metabolic reprogramming via the pyruvate dehydrogenase kinase (PDK)-pyruvate dehydrogenase complex (PDC) axis under psychological stress, exploring multidimensional impacts on semen quality.Approved by three tertiary hospitals in Central China, 557 participants were categorized into DAS and non-DAS groups based on DASS-21 scores, with clinical histories and semen samples collected. Targeted metabolomics (ATP/pyruvate/lactate), lipidomics (free fatty acids), and RT-qPCR (PDK1-4, PDH mRNA) were performed. Results revealed significantly reduced sperm motility in the DAS group (*p* < 0.01). Mechanistically, DAS upregulated PDK2/PDK4 expression and suppressed PDH expression, driving metabolic reprogramming and mitochondrial dysfunction. This study provides the first evidence that psychological stress impairs sperm motility via PDK-PDC axis dysregulation, offering novel mechanistic insights and potential therapeutic targets for male infertility.

## Highlights

• First evidence of psychological stress (depression/anxiety/stress) impairing sperm motility through mitochondrial PDK-PDC axis dysregulation.

• Mechanistic breakthrough: Stress-induced upregulation of PDK2/PDK4 and suppression of PDH drive metabolic reprogramming, leading to ATP depletion and mitochondrial dysfunction.

• Multi-omics validation: Targeted metabolomics and lipidomics reveal reduced TCA cycle intermediates (e.g., citrate, α-ketoglutarate) and elevated free fatty acids (e.g., palmitic acid) in stressed sperm.

• Clinical significance: Identifies PDK-PDC axis as a potential therapeutic target for male infertility linked to psychological stress.

## Introduction

Infertility, defined as the inability to achieve pregnancy after 12 months of unprotected intercourse, affects 8%–12% of couples worldwide, with male factors contributing to 40%–50% of cases [1]. Over the past five decades, a concerning decline in semen quality has been observed globally, with sperm concentration dropping by 52.4% and total sperm count by 59.3% in North America, Europe, and Australia [2]. This trend is associated with contemporaneous phenomena including rising obesity rates, increased exposure to environmental pollutants, and elevated psychological stress levels, suggesting underlying etiological mechanisms involving multifactorial interactions [3, 4]. Among these, psychological stress has emerged as a potential modifiable risk factor for male infertility, yet its biological underpinnings remain poorly understood [5, 6].

The burden of mental health disorders has reached epidemic proportions, with an estimated 970 million people suffering from anxiety or depressive disorders in 2022 [7]. Among men seeking fertility treatment, the prevalence of depression and anxiety is markedly elevated, ​with its prevalence being significantly higher than that in the general male population [8, 9]. Previous studies have reported inverse associations between depression and semen parameters, including semen volume, total sperm count, sperm concentration, and motility [10–12], with a mechanism potentially linked to depression-induced oxidative stress that compromises sperm quality [13, 14]. Notably, generalized anxiety disorder (GAD) has been proposed to impair testicular function through chronic stress pathways, ultimately affecting reproductive capacity [15]. These findings provide critical insights into the interplay between psychological factors and reproductive health. However, conflicting evidence suggests minimal or negligible effects of psychological stress on semen quality [16–19]. Such discrepancies may arise from methodological heterogeneity in stress assessment, population selection biases, and geographic variability, underscoring the need for standardized protocols and mechanistic investigations to elucidate causal relationships.

Sperm motility is a pivotal determinant of male fertility. Total motility refers to all motile spermatozoa (including both progressive and non-progressive motility), while progressive motility describes spermatozoa moving actively in straight lines or large circular paths at a minimum speed of 25 μm/s, according to the WHO Laboratory Manual (5th edition).The exceptional motility of spermatozoa necessitates extraordinarily high adenosine triphosphate (ATP) demands for epididymal maturation and post-ejaculatory movement [20]. Mitochondria, serving as the energy hub for sperm motility [21], generate ATP through dual metabolic pathways: glycolysis and oxidative phosphorylation (OXPHOS) [22, 23]. While numerous studies emphasize glycolysis as the primary energy source for mammalian sperm motility [24–26], accumulating evidence highlights the active involvement of fatty acid oxidation (FAO) in sperm energy metabolism and motility regulation [27–29], with FAO potentially surpassing glycolysis in dominance under specific physiological conditions [30]. The dynamic equilibrium of the mitochondrial PDK-PDC axis plays a critical role in maintaining metabolic flexibility. This axis regulates the efficiency of pyruvate conversion to acetyl-CoA, thereby directly modulating the competitive interplay between FAO and glucose metabolism. Aberrant upregulation of PDK phosphorylates and inhibits PDC activity, triggering a substrate preference shift toward FAO [31, 32]. Such metabolic reprogramming disrupts ATP homeostasis, which may constitute a central mechanism underlying reduced sperm motility. However, the molecular pathways through which psychological stress influences sperm quality via this metabolic axis remain uncharacterized, representing a critical knowledge gap at the intersection of psychobiology and reproductive medicine.

This study employed a cross-sectional design to investigate the correlation between semen quality parameters and psychological scale scores. Mechanistically, it pioneers a focused exploration of the mitochondrial PDK-PDC axis in human sperm. Through comprehensive analyses encompassing energy metabolite profiling, free fatty acid quantification, and qRT-PCR-based evaluation of PDK/PDH expression dynamics, we systematically deciphered the molecular pathways by which psychological stress disrupts sperm energy metabolism. These findings partially bridging critical knowledge gaps​ in the molecular mechanisms linking emotional distress to male infertility.

## Materials and methods

### Study design

In this multicenter cross-sectional study conducted from June 2023 to July 2024, 557 adult male volunteers were consecutively recruited from three clinical departments in Hubei Province, China: the Department of Urology at Hubei Hospital of Traditional Chinese Medicine, the Department of Reproduction at Hubei Maternal and Child Health Hospital, and the Department of Urology at Hubei Hospital of Integrated Traditional and Western Medicine. The study protocol received ethical approval from all participating institutions (Approval Nos.: HBZY2023-C10-01, 2023IEC-094, and 2023-ER-058) and was conducted in compliance with the Declaration of Helsinki. All participants provided written informed consent after receiving detailed instructions about semen sample collection procedures and study objectives, with all data processed anonymously to ensure confidentiality.

Participants were required to complete two standardized instruments. The first instrument was the 21-item Depression, Anxiety and Stress Scale (DASS-21), a validated psychometric tool consisting of three 7-item subscales that measure emotional states over the preceding week using a 4-point Likert response format (0 ="Not applicable at all"to 3 ="Very applicable"). This Chinese-adapted version has demonstrated satisfactory reliability in prior studies [33], with Cronbach's α coefficients ranging from 0.76 to 0.90 [34]. The second questionnaire collected comprehensive data through three domains: sociodemographic characteristics including age, height, body mass index (BMI), marital status, parenthood status, employment status, and economic standing;medical history encompassing psychiatric disorders, hypertension, hyperlipidemia, diabetes mellitus, hyperuricemia, and rheumatoid diseases; and modifiable lifestyle factors such as smoking habits, alcohol consumption patterns, sleep deprivation frequency, occupational overexertion, and prolonged electronic device usage.Each volunteer filled out these two questionnaires and provided a semen sample.

### Semen sample collection and analysis

Semen samples were collected through masturbation after 2–7 days of sexual abstinence, adhering to World Health Organization (WHO) guidelines. Immediately after collection into sterile containers, samples were incubated at 37 °C for 30 min to ensure complete liquefaction. Semen analysis was performed using a computer-assisted sperm analysis (CASA) system (SQA-Vision, Hamilton Thorne IVOS II) to evaluate sperm concentration (× 10⁶/mL), motility parameters (total and progressive motility, %), and Morphological characteristics. Morphology assessment followed WHO 5th edition criteria, with Diff-Quik stained smears analyzed by two independent technicians. All procedures were completed within 1 h post-collection, and inter-observer variability was controlled below 5% through standardized protocols.

### Targeted energy metabolomics

Sperm purification was performed using PureSperm density gradient centrifugation (40/80%, Nidacon), yielding high-motility sperm (> 90% viability) with > 95% purity. Purified sperm (1 × 10⁶ cells) were lysed in methanol–acetonitrile (1:1) containing 10 mM SUCCINIC ACID-D6 (Sigma-Aldrich, Cat# 488,536) internal standard, followed by ultrasonication water bath (40 kHz, 4 °C, 30 min) and protein precipitation at −20°C. After centrifugation (14,000 g, 20 min, 4 ℃), supernatants were lyophilized using a vacuum concentrator,The dried residues were resuspended in 100 μL of acetonitrile–water (1:1, v/v) prior to injection, and the resulting solution was used for LC–MS analysis. Chromatographic separation was achieved on an Agilent 1290 Infinity II UHPLC system equipped with an ACQUITY UPLC BEH Amide column (1.7 µm, 2.1 mm × 150 mm, Waters) at a column temperature of 35 °C. The mobile phase consisted of two eluents: eluent A was 50 mM ammonium acetate aqueous solution containing 1.2% ammonium hydroxide (pH 9.0), and eluent B was acetonitrile containing 1% acetylacetone. The gradient elution protocol was performed at a flow rate of 300 μL/min with an injection volume of 2 μL, following the time-dependent B-phase gradient: 70% B-phase was maintained from 0 to 1 min; B-phase linearly decreased from 70 to 60% between 1 and 10 min; B-phase further linearly decreased from 60 to 30% between 10 and 12 min; B-phase was held at 30% from 12.1 to 15 min; B-phase rapidly increased from 30% back to 70% between 15 and 15.5 min; finally, 70% B-phase was maintained from 15.5 to 22 min.A 5500 QTRAP mass spectrometer (SCIEX) operated in negative MRM mode with ESI source parameters: 450 °C, ISVF −4500 V.Quality assurance included: 1) Internal standard monitoring (CV < 15%); 2) QC samples every 10 injections (RSD < 15%); 3) Method validation showing linearity (0.01–100 μM, R^2^ > 0.99) and recovery (85–115%). Data processing used Multiquant 3.0.2 with retention time alignment.

### Targeted lipidomics-detection of free fatty acids

Sperm purification was performed using 40/80% PureSperm density gradient centrifugation (Nidacon International) with DPBS washes, yielding > 95% pure sperm with > 90% viability. For free fatty acid (FFA) analysis, 1 × 10⁶ purified sperm were homogenized in methanol-MTBE (1:1.3, 0.1% BHT) and centrifuged (12,000 g, 5 min, 4 °C). The lipid phase was derivatized with 15% BF₃-methanol containing 10 μM C17:0 internal standard (Cmass, Cat# CMS-R0542) at 60 °C for 30 min to generate fatty acid methyl esters (FAMEs). Hexane-extracted FAMEs were analyzed via GC–MS (Shimadzu GCMS-QP2020) on a DB-5MS column (30 m × 0.25 mm, 0.25 µm, Agilent Technologies) with an injection volume of 1 μL, using temperature programming: 40 °C (2 min) → 200 °C at 30°C/min → 240 °C at 10°C/min → 285 °C at 5°C/min (3 min). MS detection used electron ionization (70 eV) with ion source/quadrupole temperatures of 230°C/150°C.FFA quantification was performed using GCMS Solution software (Shimadzu) by matching fragment ions (e.g., m/z 74, 87) against the NIST 20 library (similarity > 80%). Absolute concentrations were normalized to C17:0 internal standard (CV < 10%). Metabolic pathway enrichment (KEGG ko04978) and PCA (SIMCA 14.1, UV-scaled) were conducted with significance thresholds of FDR < 0.05 and model validity criteria (R^2^X > 0.6, Q^2^ > 0.5).Quality assurance included: 1) Derivatization efficiency > 95% (spiked FFA standards); 2) Daily system suitability (Rs > 1.5, theoretical plates > 5000); 3) QC samples (pooled extracts) with RSD < 15%; 4) Method validation (linearity 0.1–100 μg/mL, R^2^ > 0.99; recovery 85–115%; intra-/inter-day precision RSD < 8%/12%); 5) Blank controls (signal < LOQ).

### Reverse transcription-quantitative PCR (RT-qPCR)

Sperm purification was performed using 40/80% PureSperm density gradient centrifugation (Nidacon International) with DPBS washes, yielding > 95% pure sperm with > 90% motility. Mitochondria were isolated from 1 × 10⁷ sperm using a commercial kit (Thermo Scientific, #89,874) through differential centrifugation: initial lysis (700 g, 10 min, 4 °C) to remove debris, followed by mitochondrial enrichment (12,000 g, 15 min, 4 °C).Total RNA was extracted using a Servicebio kit (#G3640), with quality verified by A260/A280 ratios (1.8–2.0) and agarose gel electrophoresis. First-strand cDNA synthesis was performed with 1 μg RNA using a Servicebio reverse transcription kit (#G3337) under standard conditions: 42 °C for 20 min, 85 °C for 5 s.Quantitative PCR utilized SYBR Green master mix (Servicebio, #G3326) with gene-specific primers (Table 1, designed by Wuhan Cyvier Biotechnology) in triplicate reactions. Cycling parameters: 95 °C for 30 s, 40 cycles of 95°C/15 s and 60°C/30 s. Relative gene expression was calculated via 2 − ΔΔCT method using GAPDH for normalization.Quality control included: 1) RNA integrity verification; 2) primer specificity confirmation through melt curve analysis; 3) technical triplicates (CV < 5%); 4) NTC controls.

**Table 1:** Primer Sequences

Gene	Species	Primer Sequence (5'−3')	Fragment Length (bp)
PDK1	Human	S: TGTGAAGATGAGTGACCGAGGAG	216
		A: GCATCTGTCCCGTAACCCTCTA	
PDK2	Human	S: TACCTCAGCCGCATCTCCAT	131
		A: TTGACCACCTCAGAGACGTTG	
PDK3	Human	S: GATAATTTACTTAACCGCCCTTCAG	230
		A: GTGCTAATGAAAGGATCAAACCC	
PDK4	Human	S: AAGCCCAGATGACCAGAAAGC	210
		A: TGGTTCATCAGCATCCGAGTAGA	
PDH	Human	S: CGAATTGGAATCCCAGTCAGAAG	71
		A: AGTTGAGTTGGTGCTGGCATG	
GAPDH	Human	S: GGAAGCTTGTCATCAATGGAAATC	168
		A: TGATGACCCTTTTGGCTCCC	

### Statistical analysis

Statistical analyses were performed using IBM SPSS Statistics 25.0 and RStudio (version 4.3.1), with data visualization conducted in GraphPad Prism 8. The normality of continuous variables was assessed by Shapiro–Wilk tests (α = 0.05). Normally distributed data were analyzed using independent t-tests, while non-normally distributed data were evaluated with Mann–Whitney U tests. Statistical significance was defined as two-tailed *p* < 0.05.

## Results

This study enrolled 557 healthy male volunteers with a mean age of 35.02 ± 8.84 years. Table 2 summarizes sociodemographic characteristics and lifestyle factors. Notably, 30.16% reported smoking, 41.65% alcohol consumption, 61.40% frequent night-time activity, 32.68% occupational overexertion, and 85.82% prolonged electronic device usage.

**Table 2:** Descriptive analysis of the questionnaire data from male volunteers. Data are presented as mean ± standard deviation (SD) or percentages

**Parameter**		**N**
Age (years)	35.02 ± 8.84	557
Height (m)	1.73 ± 0.05	555
Weight (kg)	72.15 ± 9.59	555
BMI (kg/m2)	23.99 ± 3.20	555
Get married		
No	29.8	166
Yes	70.2	391
Have children		
No	50.99	284
Yes	49.01	273
Have a regular job		
No	12.93	72
Yes	87.07	485
There are other sources of income		
No	81.33	453
Yes	18.67	104
Have a history of mental illness		
No	97.85	545
Yes	2.15	12
High blood pressure		
No	91.74	511
Yes	8.26	46
High blood fat		
No	91.35	507
Yes	8.65	48
Diabetes		
No	97.48	541
Yes	2.52	14
Hyperuricemia		
No	91.53	508
Yes	8.47	47
Internal rheumatism		
No	98.92	549
Yes	1.08	6
Smoking status		
No	69.84	389
Yes	30.16	168
Drinking status		
No	58.35	325
Yes	41.65	232
Stay up late		
No	38.6	215
Yes	61.4	342
Overwork		
No	67.32	375
Yes	32.68	182
Use Electronics for a long time		
No	14.18	79
Yes	85.82	478

Participants were stratified into groups based on DASS-21 thresholds (depression: 0–9; anxiety: 0–7; stress: 0–14), with psychological distress observed in 38.60% (depression), 56.73% (anxiety), and 27.10% (stress) of participants. Severity stratification is detailed in Table 3.

**Table 3:** Prevalence of depression, anxiety, and stress among male volunteers. Data are expressed as percentages

	**N(%)**		
**DAS level**	**Depression**	**Anxiety**	**Stress**
Normal	342 (61.40)	241 (43.27)	406 (72.90)
Mild	99 (17.77)	75 (13.46)	71 (12.74)
Moderate	81 (14.54)	163 (29.26)	48 (8.62)
Severe	26 (4.67)	46 (8.26)	28 (5.02)
Extremely severe	9 (1.62)	32 (5.75)	4 (0.72)

Semen analysis revealed the following parameters (mean ± SD): concentration 57.35 ± 23.95 × 10⁶/mL, total motility 45.94 ± 6.72%, progressive motility 36.95 ± 5.74%, and normal Morphology 7.64 ± 3.46% (Table 4).

**Table 4:** Descriptive analysis of the routine semen parameters of male volunteers. Data are presented as mean ± standard deviation (SD)

**Semen quality parameters**		**N**
Concentration (× 10^6^/mL)	57.35 ± 23.95	557
Total motility (Progressive and non-progressive, %)	45.94 ± 6.72	557
Progressive motility (%)	36.95 ± 5.74	557
Normal morphological sperm (%)	7.64 ± 3.46	557

### The impact of depression, anxiety, and stress on routine semen quality parameters

**Fig. 1:**
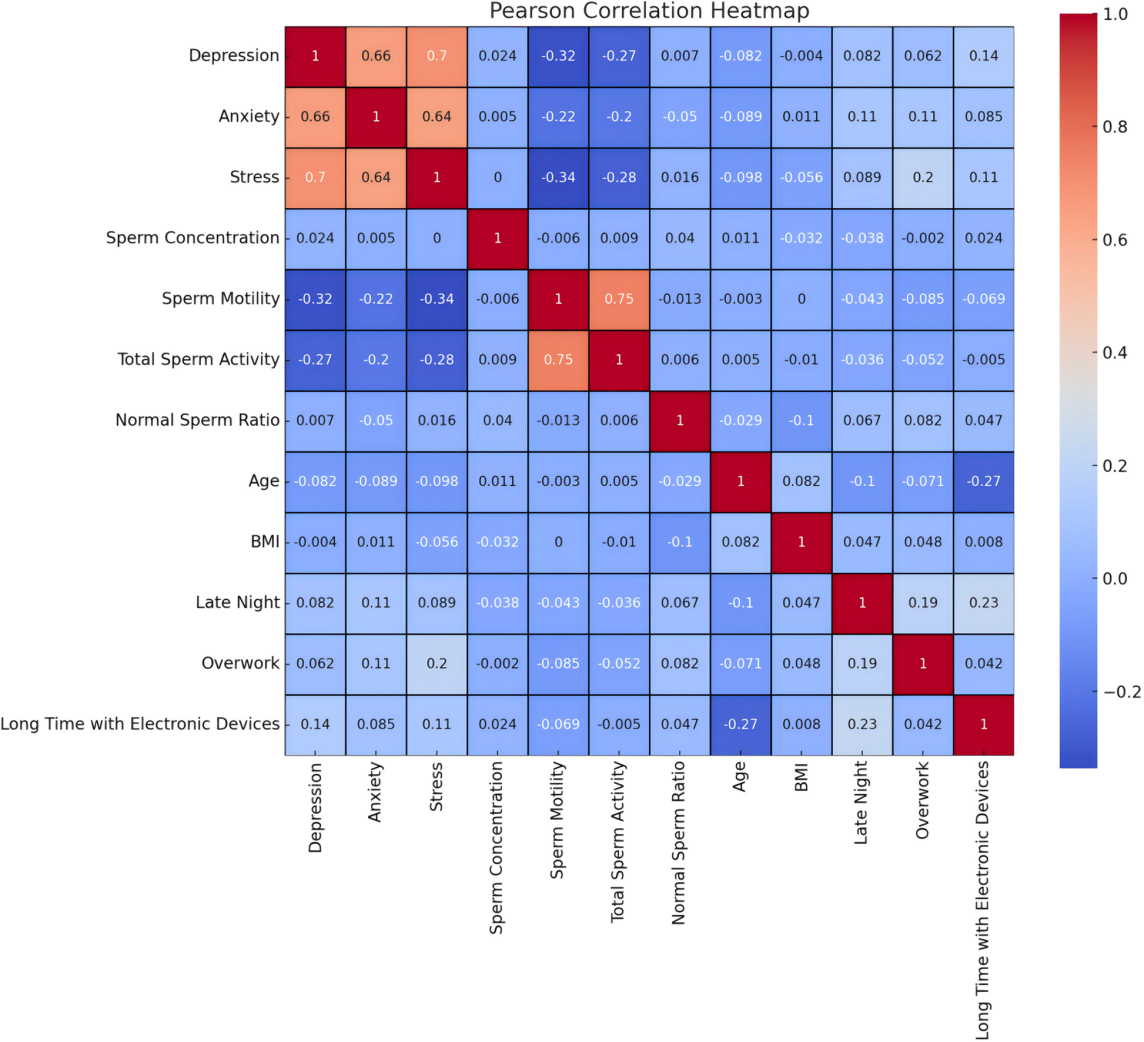
Pearson correlation matrix of psychological distress scores and semen parameters. The heatmap illustrates pairwise correlations between depression, anxiety, stress scores (DASS-21 subscales), age, BMI, and semen quality parameters. Color intensity reflects the magnitude of Pearson's correlation coefficient (|r|), with red indicating positive correlations and blue negative correlations (see scale bar). Coefficients range from −1 (perfect inverse) to + 1 (perfect direct relationship)

Bivariate correlations between psychological distress scores (depression, anxiety, stress), age, BMI, and semen parameters were assessed using Pearson's r, with correlation patterns visualized through a heatmap (Fig. 1).

**Fig. 2:**
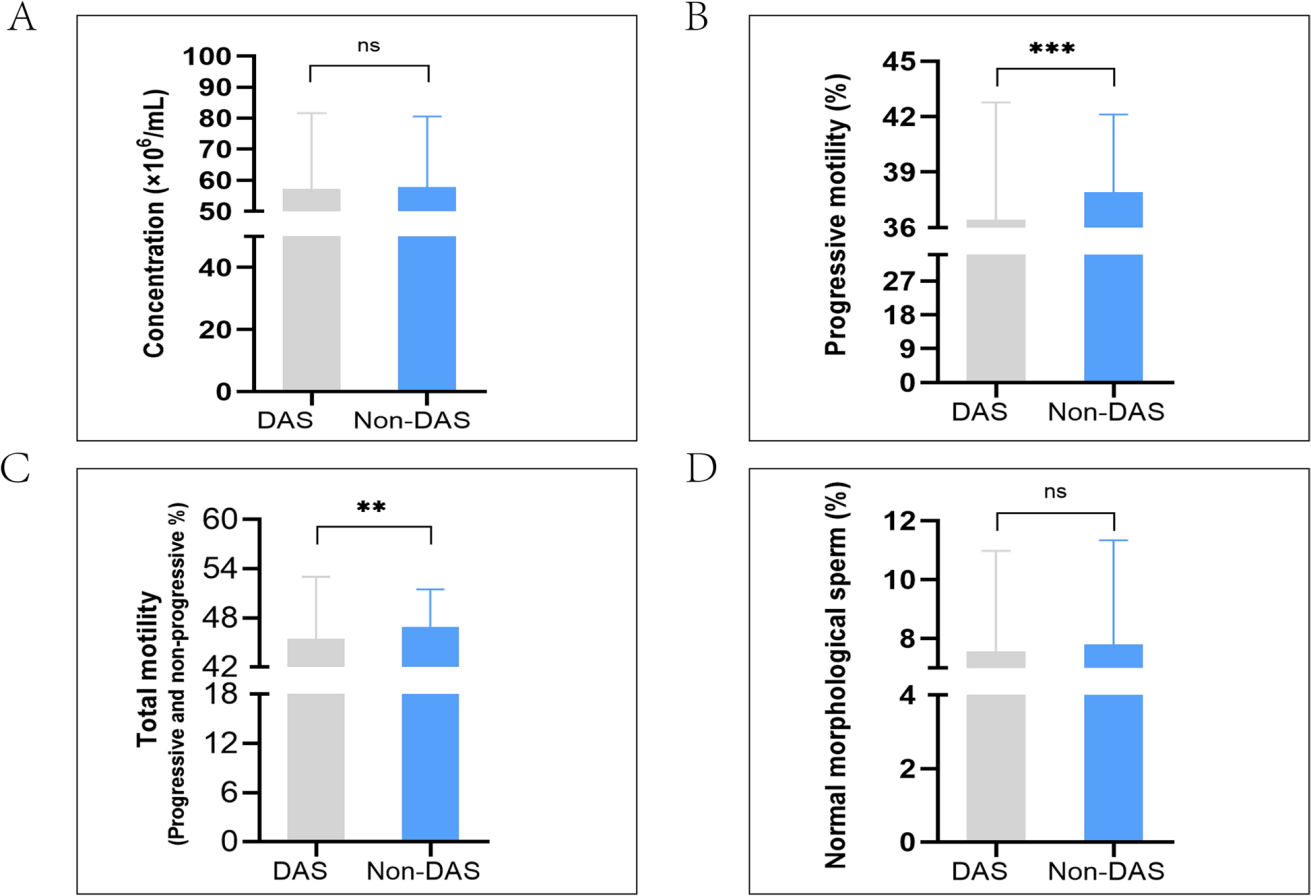
Comparative analysis of semen parameters between DAS and Non-DAS groups. Bar plots show (**A**) sperm concentration, (**B**) progressive motility, (**C**) total motility, and (**D**) normal morphology. Data are presented as mean ± SD (*n* = 557), with significance levels denoted as **** p < 0.001,** p < 0.01,* *p* < 0.05, ns (not significant)

The analysis revealed that depression, anxiety, and stress exerted significant negative effects on semen quality. Specifically, the DAS group exhibited significantly lower progressive motility (*p* < 0.001) and total motility (*p* = 0.002) compared to the Non-DAS group, as detailed in Fig. 2 and Table 5. The DAS group showed no significant differences in sperm concentration or normal morphology compared to the Non-DAS group (Table 5).

**Table 5:** Comparative analysis of semen parameters between DAS and Non-DAS groups

	**M(P**_**25**_**, P**_**75**_**)**		***p-Value***
**Parameter**	**Non-DAS**	**DAS**	
	**(N = 197)**	**(*N* = 360)**	
Age (years)	34.000(30.000,39.000)	33.000(29.000,38.750)	0.175
Height (m)	1.730(1.700,1.750)	1.730(1.700,1.760)	0.167
Weight (kg)	71.000(65.000,76.000)	71.000(65.375,78.000)	0.531
BMI (kg/m2)	23.700(22.150,25.900)	23.900(22.200,25.575)	0.892
Concentration (× 10^6^/mL)	56.840(38.190,77.615)	56.595(35.625,78.665)	0.707
Progressive motility (%)	37.610(34.760,40.815)	35.695(32.073,40.343)	< 0.001***
Total motility (Progressive and non-progressive, %)	45.810(43.125,50.330)	45.205(40.032,50.020)	0.002**
Normal morphological sperm (%)	8.000(5.000,11.000)	8.000(5.000,10.000)	0.422

^†^Data presented as median (IQR); Mann–Whitney U test with *** p < 0.001; **p < 0.01;* *p* < 0.05

### Depression, anxiety, and stress impair glucose metabolism, disrupt TCA Cycle, and reduce electron transport chain efficiency

**Fig. 3:**
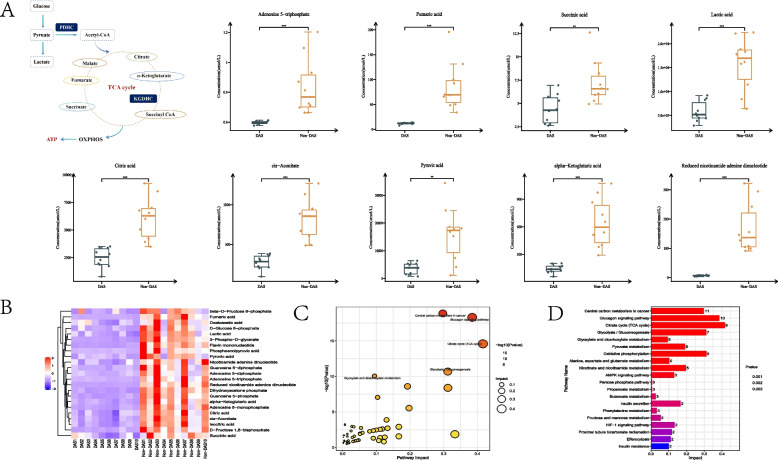
Comparative analysis of energy metabolism between DAS and Non-DAS groups. **A** Schematic of the TCA cycle with quantitative metabolite levels in glycolysis, TCA cycle, and oxidative phosphorylation. **B** Hierarchical clustering of differential metabolites (red: upregulation; purple: downregulation). **C** KEGG pathway enrichment map. **D** Top 10 significantly enriched metabolic pathways. Data expressed as mean ± SD (*n* = 10 biological replicates per group). ***p < 0.001, **p < 0.01,*p < 0.05

Targeted metabolomic analysis identified 26 key metabolites involved in glycolysis, the tricarboxylic acid (TCA) cycle, and oxidative phosphorylation. The DAS group exhibited significantly reduced ATP levels (*p* < 0.001) and downregulation of 22 metabolites compared to controls (Fig. 3A). Critical glycolytic intermediates, including dihydroxyacetone phosphate (DHAP), fructose-1,6-bisphosphate (F1,6BP), fructose-6-phosphate (F6P), and pyruvate, were markedly decreased (Fig. 3B). Concurrently, TCA cycle inhibition was evidenced by reduced citrate, α-ketoglutarate, oxaloacetate, and fumarate levels (all *p* < 0.05). Furthermore, electron transport chain (ETC) dysfunction was observed through diminished NADH and FMN (flavin mononucleotide) concentrations (*p* < 0.05).KEGG pathway enrichment analysis of the 22 differentially expressed metabolites (Fig. 3C-D) revealed significant perturbations in core metabolic processes: central carbon metabolism, glucagon signaling, TCA cycle, glycolysis/gluconeogenesis, pyruvate metabolism, and oxidative phosphorylation.

### Depression, anxiety, and stress induce lipid metabolic imbalance through free fatty acid accumulation

**Fig. 4:**
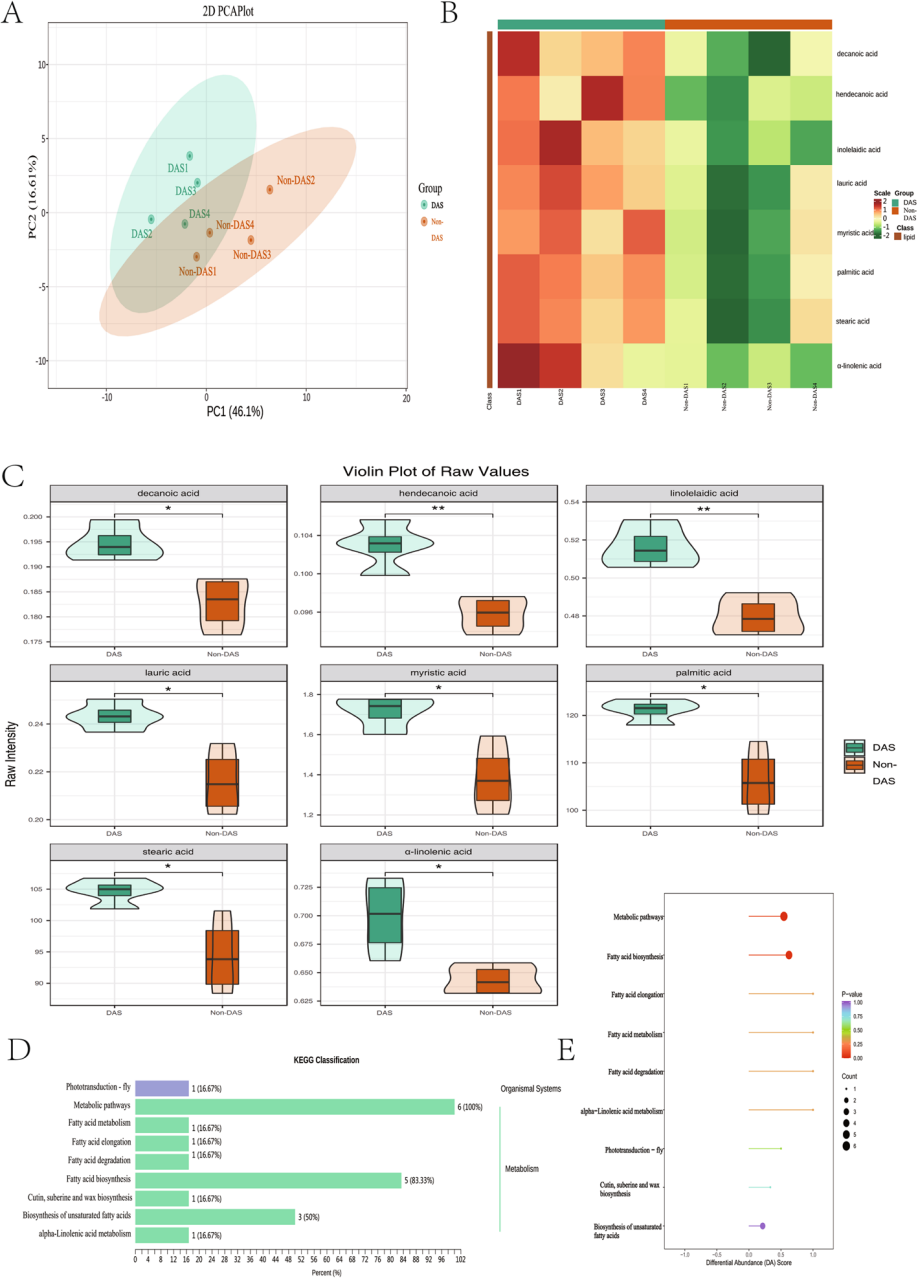
Lipid metabolic alterations in the DAS group (**A**) PCA score plot showing group separation (DAS vs. Non-DAS). (**B**) Heatmap of differentially expressed FFAs (red: upregulation; green: downregulation). **C** Violin plots quantifying FFA levels (***p < 0.001, **p < 0.01,*p < 0.05). (D) KEGG classification showing pathway-to-metabolite ratios. **E** Pathway impact analysis with differential abundance scores. Data expressed as mean ± SD (*n* = 4 biological replicates)

Targeted lipidomic analysis via GC–MS/MS identified 31 free fatty acids (FFAs). Principal component analysis (PCA) indicated a partial separation of lipid metabolic profiles between the DAS and Non-DAS groups, though the 95% confidence ellipses overlapped (Fig. 4A). Eight FFAs exhibited significant differential expression (P < 0.05): decanoic acid (C10:0), hendecanoic acid (C11:0), linolelaidic acid (trans-C18:2n6), lauric acid (C12:0), myristic acid (C14:0), palmitic acid (C16:0), stearic acid (C18:0), and α-linolenic acid (C18:3n3), all showing upregulation in the DAS group (Fig. 4B-C).KEGG pathway enrichment analysis demonstrated these metabolites were predominantly involved in lipid metabolic pathways, fatty acid biosynthesis and unsaturated fatty acid biosynthesis (Fig. 4D-E).

**Fig. 5:**
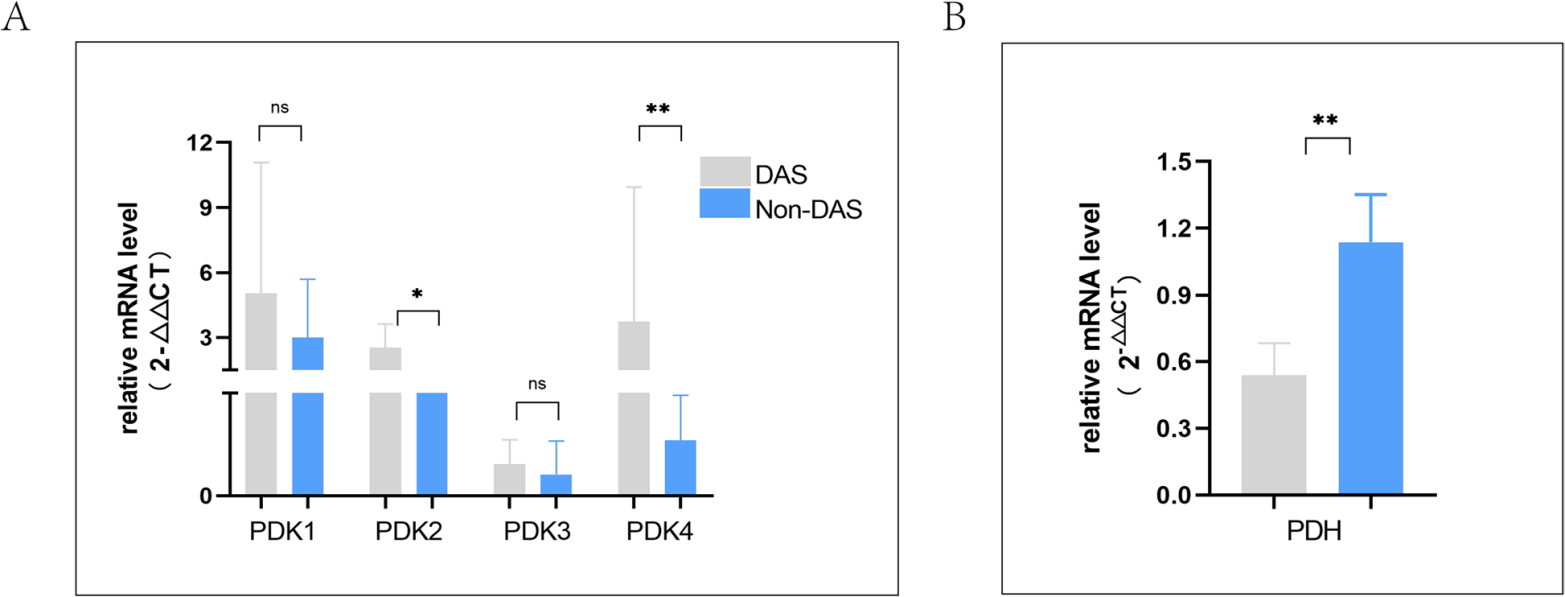
Expression profiles of PDK isoforms and PDH between DAS and Non-DAS groups (**A**) Relative mRNA levels of PDK1, PDK2, PDK3, and PDK4. (B) PDH mRNA expression was significantly reduced in the DAS group. Data expressed as mean ± SD (n = 8 biological replicates). ***p < 0.001, **p < 0.01,*p < 0.05, ns (not significant)

RT-qPCR analysis revealed differential expression of pyruvate dehydrogenase kinase (PDK) isoforms 1–4 and pyruvate dehydrogenase complex (PDH) in sperm mitochondria between DAS and Non-DAS groups. PDK2 and PDK4 expressions were significantly upregulated in the DAS group (*p* < 0.05 and *p* < 0.01, respectively), whereas PDK1 and PDK3 showed non-significant upward trends(Fig. 5A). Importantly, PDH expression was significantly downregulated in the DAS group (*p* < 0.01), suggesting potential impairment of pyruvate oxidation capacity (Fig. 5B).

## Discussion

The global decline in human semen quality over recent decades [35–37] has prompted urgent investigations into modifiable risk factors. Psychological stress, as a potentially reversible contributor to male infertility, remains mechanistically controversial. By integrating psychometric evaluation (DASS-21), targeted energy metabolomics, lipidomics, and qPCR analysis, this study systematically demonstrates the correlation between depression/anxiety/stress (DAS) severity and impaired sperm motility, while pioneering the identification of the mitochondrial PDK-PDC axis as the molecular hub underlying this association. This breakthrough parallels discoveries in neurodegenerative diseases, where chronic stress-induced metabolic dysregulation similarly compromises neuronal bioenergetics [38], It suggests the existence of conserved stress—response pathways across different tissues, highlighting the universality of stress—related metabolic disruptions in biological systems..

**Fig. 6:**
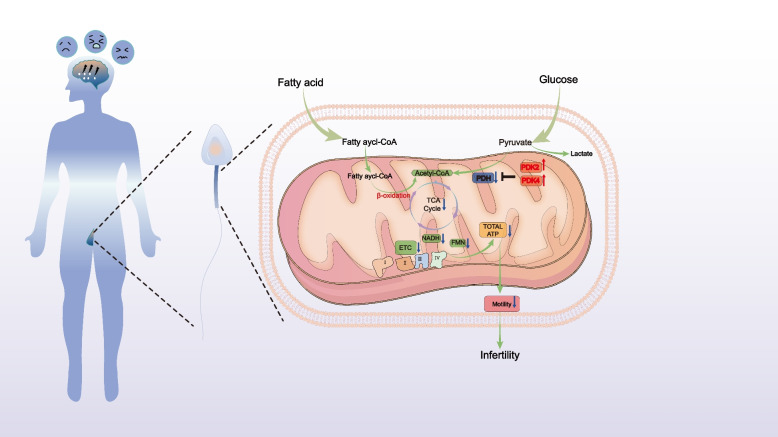
Overview of metabolic reprogramming in male sperm mitochondria under depression, anxiety, and stress:Compared with those without emotional distress, the expression of PDK2 and PDK4 in male sperm mitochondria was up-regulated under depression, anxiety and stress, which inhibited PDH, inhibited glucose metabolism pathway, blocked TCA cycle and decreased ETC efficiency, fatty acid oxidation is forced up and total ATP production is reduced

The mitochondrial PDK-PDC axis serves as a central regulatory node in sperm energy metabolism, coordinating glucose and fatty acid oxidation by dynamically balancing the activity of the PDH. This study provides the first experimental evidence that psychological stress hijacks this regulatory system through isoform-specific mechanisms (Fig. 6): overexpression of PDK2 and PDK4 in the DAS group sperm drives PDH suppression, sequestering pyruvate in the cytosol and blocking its conversion to acetyl-CoA. This directly depletes key TCA cycle intermediates, including citrate and α-ketoglutarate. Concurrently, diminished NADH and FMN availability reduces electron transport chain (ETC) efficiency, ultimately leading to decreased ATP synthesis. Termed"dual-engine shutdown"(dual inhibition of glycolysis and TCA cycle), this phenomenon fundamentally differs from the Warburg effect in cancer cells [39].While both cancer cells and stressed sperm undergo metabolic reprogramming, their objectives diverge fundamentally. The Warburg effect fuels cancer proliferation by shunting glycolytic intermediates to biosynthesis despite functional mitochondria [39]. In contrast, DAS-stressed sperm face systemic energy failure due to mitochondrial dysfunction: glycolysis suppression (↓pyruvate/F1,6BP), TCA cycle blockade (↓citrate/α-ketoglutarate), and failed compensatory β-oxidation (Fig. 3–4). This"dual-engine shutdown"arises from sperm's unique bioenergetic constraints—unlike somatic cells, sperm lack biosynthetic demands but require extreme ATP flux for flagellar motility [20], making them uniquely vulnerable to PDK-PDC dysregulation. KEGG pathway enrichment analysis further confirms significant enrichment of differentially expressed metabolites in core energy production pathways (TCA cycle and oxidative phosphorylation), indicating systemic metabolic remodeling under psychological stress, highlighting the complexity of the metabolic response to stress in sperm cells.

Faced with glycolytic collapse, sperm attempt to compensate for the energy deficit through β-oxidation—a process evidenced by significant upregulation of eight free fatty acids (FFAs), including palmitic acid (C16:0) and stearic acid (C18:0), as revealed by lipidomic profiling. However, this pseudo-compensatory response fails to restore ATP depletion and may exacerbate reactive oxygen species (ROS) generation, triggering oxidative stress that damages intracellular organelles and impairs sperm motility and function [40], thereby establishing a vicious cycle of metabolic disturbance → oxidative damage → energy depletion. This maladaptive cascade resembles non-alcoholic fatty liver disease (NAFLD), in which excessive FFAs induce mitochondrial dysfunction through incomplete oxidation [41]. Furthermore, excessive accumulation of long-chain fatty acids (LCFAs) may directly disrupt sperm plasma membrane fluidity through lipotoxicity [42], further compromising motility, as the integrity and fluidity of the plasma membrane are crucial for sperm—egg interaction and the sperm's ability to swim towards the egg.

From an evolutionary perspective, the PDK-PDC axis originally evolved as an adaptive mechanism to combat nutritional deprivation—suppressing glucose oxidation while enhancing lipolysis to sustain energy supply during starvation [43]. However, chronic psychological stress pathologically activates this conserved pathway, trapping sperm in a"metabolic gridlock."Notably, although PDK1 and PDK3 expression trends did not reach statistical significance, their upregulation suggests progressive isoform-specific adaptation to prolonged stress. These molecular compensatory attempts paradoxically exacerbate metabolic dysregulation, mirroring the neurodegenerative consequences of Tau hyperphosphorylation in Alzheimer's disease [44], ultimately leading to irreversible energetic failure in sperm, highlighting the delicate balance of sperm metabolism and the detrimental effects of chronic stress on this balance.

This study presents the first systematic demonstration of the molecular mechanism by which psychological stress impairs sperm motility through mitochondrial PDK-PDC axis-mediated metabolic reprogramming. While prior studies have established significant negative correlations between psychological stress and semen parameters (e.g., sperm concentration and motility) [6, 10, 45–47] their mechanistic focus has largely centered on hormonal imbalances (e.g., testosterone suppression [48, 49] or oxidative stress pathways [50, 51]. Our findings represent a breakthrough by positioning metabolic regulation as the core mechanism, offering novel insights into stress-associated infertility. Through integrated multi-omics analysis, this study delineates the specific pathway by which psychological stress (DAS) drives metabolic reprogramming via the PDK-PDC axis. DAS induces isoform-specific overexpression of mitochondrial PDK2 and PDK4 in sperm, suppressing PDH activity and blocking pyruvate conversion to acetyl-CoA, thereby restricting TCA cycle flux. Although lipidomic profiling detected free fatty acid accumulation (e.g., palmitic acid, C16:0), indicative of compensatory β-oxidation activation, impaired oxidative phosphorylation efficiency (evidenced by significant reductions in NADH and FMN levels) reduced ATP synthesis, ultimately resulting in the loss of sperm progressive motility (Fig. 6). This"dual-engine shutdown"mechanism—distinct from the energy-compensatory Warburg effect in cancer [39]—provides a metabolic explanation for the global epidemiological overlap between semen quality decline and psychological disorders.This metabolic specificity explains the differential vulnerability of semen parameters to psychological stress, prompting further mechanistic exploration into compensatory adaptations.

Although β-oxidation partially compensates for glycolytic failure (as evidenced by FFA accumulation), its inefficiency in sperm mitochondria [52] prompts the exploration of alternative pathways. Drosophila studies indicate that ketone bodies can serve as emergency fuels for neurons to compensate for β-oxidation deficiency [53], suggesting a rationale for exploring their compensatory potential in germ cells. PDK inhibitors (e.g., DCA) significantly improve cellular function in metabolic stress models such as renal injury [54], implying therapeutic potential for sperm energy metabolism disorders. Notably, glucocorticoid receptor (GR) activation directly upregulates PDK4 transcription [55], thereby inhibiting PDH activity and driving metabolic dysfunction—a mechanism linking HPA axis hyperactivity to sperm bioenergetic failure.

Notably, DAS was associated with selective impairment of sperm motility, while concentration and morphology remained unaffected (Table 5), suggesting distinct pathogenic mechanisms. This selectivity may arise because motility primarily depends on mitochondrial bioenergetics—potentially disrupted by PDK-PDC axis dysregulation—whereas concentration relies on spermatogenesis modulated by complex testosterone responses to stress (e.g., chronic suppression versus acute elevation) [56]. Morphogenesis during spermiogenesis involves structural processes that appear less vulnerable to metabolic ATP depletion [57], and compensatory glucocorticoid signaling may upregulate androgen receptors to partially preserve spermatogenesis [58]. Future studies measuring serial hormonal dynamics are needed to validate these hypotheses.

Although our integrated model addresses compensatory mechanisms, several limitations warrant consideration. First, the cohort was exclusively recruited from three hospitals in Hubei Province, China, which may restrict the generalizability of our findings to broader populations. Although we collected basic demographic data, detailed information on participants'geographic origins and long-term residence was not available, precluding subgroup analyses of regional influences.Second, the cross-sectional design inherently limits causal inference between psychological stress and sperm quality impairment. Future longitudinal studies tracking stress dynamics and semen parameters over time, or experimental models (e.g., stress-induced animal studies), are needed to establish causality.Third, while we focused on the mitochondrial PDK-PDC axis, other metabolic pathways (e.g., glycolytic flux, oxidative phosphorylation) may also mediate stress-induced sperm damage and warrant investigation.

## Conclusions

This study delineates the molecular mechanism by which depression/anxiety/stress impairs sperm function through mitochondrial PDK-PDC axis-mediated metabolic reprogramming. We confirm that stress-induced PDK2/4 overexpression and PDH suppression establish a"dual metabolic blockade"of glucose and lipid utilization, depleting ATP production and motility. The observed metabolic gridlock differs fundamentally from the Warburg effect, highlighting sperm-specific metabolic vulnerability under stress. Our pioneering integration of evolutionary biology into reproductive medicine reveals that PDK-PDC axis dysregulation represents pathological hijacking of a conserved stress-response pathway. These findings establish a theoretical foundation for developing non-invasive diagnostics (e.g., PDK4 expression levels) and precision therapies (e.g., isoform-specific PDK inhibitors), advancing male infertility management from phenomenological observation to mechanism-targeted regulation.

## Data Availability

The datasets generated during and/or analyzed during the current study are not publicly available but may be made available upon reasonable request to the corresponding author.

## Abbreviations

PDK: Pyruvate dehydrogenase kinase
PDC: Pyruvate dehydrogenase complex
DASS: Depression anxiety stress stable
PDH: Pyruvate dehydrogenase
ATP: Adenosine triphosphate
BMI: Body mass index
SE: Standard deviation
PR: Progressive motility
NP: Non-progressive motility
TCA: Tricarboxylic acid cycle
NADH: Nicotinamide adenine dinucleotide
FMN: Flavin monoucleotide
KEGG: Kyoto encyclopedia of genes and genomes

## References

[1] Vander Borght M, Wyns C. Fertility and infertility: Definition and epidemiology. Clin Biochem. 2018;62:2–10. 29555319 10.1016/j.clinbiochem.2018.03.012

[2] Levine H, Jorgensen N, Martino-Andrade A, Mendiola J, Weksler-Derri D, Mindlis I, Pinotti R, Swan SH. Temporal trends in sperm count: a systematic review and meta-regression analysis. Hum Reprod Update. 2017;23(6):646–59. 28981654 10.1093/humupd/dmx022 PMC6455044

[3] Gore AC, Chappell VA, Fenton SE, Flaws JA, Nadal A, Prins GS, Toppari J, Zoeller RT. EDC-2: The Endocrine Society’s Second Scientific Statement on Endocrine-Disrupting Chemicals. Endocr Rev. 2015;36(6):E1–150. 26544531 10.1210/er.2015-1010 PMC4702494

[4] Skakkebaek NE, Lindahl-Jacobsen R, Levine H, Andersson A, Jorgensen N, Main KM, Lidegaard O, Priskorn L, Holmboe SA, Brauner EV, Almstrup K, Franca LR, Znaor A, Kortenkamp A, Hart RJ, Juul A. Environmental factors in declining human fertility. Nat Rev Endocrinol. 2022;18(3):139–57. 34912078 10.1038/s41574-021-00598-8

[5] Ilacqua A, Izzo G, Emerenziani GP, Baldari C, Aversa A. Lifestyle and fertility: the influence of stress and quality of life on male fertility. Reprod Biol Endocrin. 2018;16(1):115. 10.1186/s12958-018-0436-9 PMC6260894 30474562

[6] Janevic T, Kahn LG, Landsbergis P, Cirillo PM, Cohn BA, Liu X, Factor-Litvak P. Effects of work and life stress on semen quality. Fertil Steril. 2014;102(2):530–8. 24856463 10.1016/j.fertnstert.2014.04.021 PMC4382866

[7] Collaborators GMD. Global, regional, and national burden of 12 mental disorders in 204 countries and territories, 1990–2019: a systematic analysis for the Global Burden of Disease Study 2019. Lancet Psychiat. 2022;9(2):137–50. 10.1016/S2215-0366(21)00395-3 PMC8776563 35026139

[8] Yang B, Zhang J, Qi Y, Wang P, Jiang R, Li H. Assessment on Occurrences of Depression and Anxiety and Associated Risk Factors in the Infertile Chinese Men. Am J Mens Health. 2017;11(3):767–74. 28413943 10.1177/1557988317695901 PMC5675225

[9] Mor M, Jayaseelan V, Kattimani S, Thyagaraju C, Kubera NS, Duraiswamy M. Depression, anxiety, stress, and coping among men with infertility seeking treatment at a tertiary care hospital in South India: A mixed-method study. Indian J Psychiat. 2024;66(12):1131–8. 10.4103/indianjpsychiatry.indianjpsychiatry_504_24 PMC11758972 39867237

[10] Bhongade MB, Prasad S, Jiloha RC, Ray PC, Mohapatra S, Koner BC. Effect of psychological stress on fertility hormones and seminal quality in male partners of infertile couples. Andrologia. 2015;47(3):336–42. 24673246 10.1111/and.12268

[11] Zorn B, Auger J, Velikonja V, Kolbezen M, Meden-Vrtovec H. Psychological factors in male partners of infertile couples: relationship with semen quality and early miscarriage. Int J Androl. 2008;31(6):557–64. 17651396 10.1111/j.1365-2605.2007.00806.x

[12] Zou P, Wang X, Sun L, Chen Q, Yang H, Zhou N, Chen H, Zhang G, Ling X, Wang Z, Gao J, Mo M, Huang L, Peng K, Chen S, Cui Z, Liu J, Ao L, Cao J. Semen Quality in Chinese College Students: Associations With Depression and Physical Activity in a Cross-Sectional Study. Psychosom Med. 2018;80(6):564–72. 29794946 10.1097/PSY.0000000000000595

[13] Pandya CD, Howell KR, Pillai A. Antioxidants as potential therapeutics for neuropsychiatric disorders. Prog Neuro-Psychoph. 2013;46:214–23. 10.1016/j.pnpbp.2012.10.017 PMC3615047 23123357

[14] Lindqvist D, Dhabhar FS, James SJ, Hough CM, Jain FA, Bersani FS, Reus VI, Verhoeven JE, Epel ES, Mahan L, Rosser R, Wolkowitz OM, Mellon SH. Oxidative stress, inflammation and treatment response in major depression. Psychoneuroendocrino. 2017;76:197–205. 10.1016/j.psyneuen.2016.11.031 PMC5272818 27960139

[15] Pan Y, Wang S, Kang J, Cao T, Liu J, Zhang L, Niu S, Liu X. Association between generalized anxiety symptoms and semen quality in infertile men: A multicentre study in North China. Andrologia. 2022;54(8): e14449. 35491407 10.1111/and.14449

[16] Gurhan N, Akyuz A, Atici D, Kisa S. Association of depression and anxiety with oocyte and sperm numbers and pregnancy outcomes during in vitro fertilization treatment. Psychol Rep. 2009;104(3):796–806. 19708407 10.2466/PR0.104.3.796-806

[17] Roopnarinesingh R, Keane D, Harrison R. Detecting mood disorders in men diagnosed with cancer who seek semen cryopreservation: a chance to improve service. Ir Med J 2003, 96(4): 104, 106–107. 12793470

[18] Coward RM, Stetter C, Kunselman A, Trussell JC, Lindgren MC, Alvero RR, Casson P, Christman GM, Coutifaris C, Diamond MP, Hansen KR, Krawetz SA, Legro RS, Robinson RD, Smith JF, Steiner AZ, Wild RA, Zhang H, Santoro N. Fertility Related Quality of Life, Gonadal Function and Erectile Dysfunction in Male Partners of Couples with Unexplained Infertility. J Urology. 2019;202(2):379–84. 10.1097/JU.0000000000000205 PMC6686175 30835629

[19] Hjollund NHI, Bonde JPE, Henriksen TB, Giwercman A, Olsen J. Reproductive effects of male psychologic stress. Epidemiology. 2004;15(1):21–7. 14712143 10.1097/01.ede.0000100289.82156.8b

[20] Piomboni P, Focarelli R, Stendardi A, Ferramosca A, Zara V. The role of mitochondria in energy production for human sperm motility. Int J Androl. 2012;35(2):109–24. 21950496 10.1111/j.1365-2605.2011.01218.x

[21] Boguenet M, Bouet P, Spiers A, Reynier P, May-Panloup P. Mitochondria: their role in spermatozoa and in male infertility. HUM REPROD UPDATE. 2021;27(4):697–719. 33555313 10.1093/humupd/dmab001

[22] Ferramosca A, Zara V. Bioenergetics of mammalian sperm capacitation. BIOMED RES INT. 2014;2014: 902953. 24791005 10.1155/2014/902953 PMC3984864

[23] Buffone MG, Ijiri TW, Cao W, Merdiushev T, Aghajanian HK, Gerton GL. Heads or tails? Structural events and molecular mechanisms that promote mammalian sperm acrosomal exocytosis and motility. MOL REPROD DEV. 2012;79(1):4–18. 22031228 10.1002/mrd.21393 PMC3240700

[24] Mukai C, Travis AJ. What sperm can teach us about energy production. Reprod Domest Anim 2012, 47 Suppl 4(0 4): 164–169. 10.1111/j.1439-0531.2012.02071.x PMC3727149 22827366

[25] Storey BT. Mammalian sperm metabolism: oxygen and sugar, friend and foe. Int J Dev Biol. 2008;52(5–6):427–37. 18649255 10.1387/ijdb.072522bs

[26] Turner RM. Moving to the beat: a review of mammalian sperm motility regulation. Reprod Fert Develop. 2006;18(1–2):25–38. 10.1071/rd05120 16478600

[27] Asghari A, Marashi S, Ansari-Pour N. A sperm-specific proteome-scale metabolic network model identifies non-glycolytic genes for energy deficiency in asthenozoospermia. SYST Biol Reprod Med. 2017;63(2):100–12. 28085499 10.1080/19396368.2016.1263367

[28] Paiva C, Amaral A, Rodriguez M, Canyellas N, Correig X, Ballesca JL, Ramalho-Santos J, Oliva R. Identification of endogenous metabolites in human sperm cells using proton nuclear magnetic resonance ((1) H-NMR) spectroscopy and gas chromatography-mass spectrometry (GC-MS). Andrology-US. 2015;3(3):496–505. 10.1111/andr.12027 25854681

[29] Chauvin T, Xie F, Liu T, Nicora CD, Yang F, Camp DGN, Smith RD, Roberts KP. A systematic analysis of a deep mouse epididymal sperm proteome. Biol Reprod. 2012;87(6):141. 23115268 10.1095/biolreprod.112.104208 PMC4435428

[30] Amaral A, Lourenco B, Marques M, Ramalho-Santos J. Mitochondria functionality and sperm quality. Reproduction. 2013;146(5):R163–74. 23901129 10.1530/REP-13-0178

[31] Foster DW. Malonyl-CoA: the regulator of fatty acid synthesis and oxidation. J Clin Invest. 2012;122(6):1958–9. 22833869 10.1172/JCI63967 PMC3366419

[32] Sugden MC, Kraus A, Harris RA, Holness MJ. Fibre-type specific modification of the activity and regulation of skeletal muscle pyruvate dehydrogenase kinase (PDK) by prolonged starvation and refeeding is associated with targeted regulation of PDK isoenzyme 4 expression. Biochem J 2000, 346 Pt 3(Pt 3): 651–657. PMC1220897 10698691

[33] Hou T, Zhang F, Mao X, Deng G. Chronotype and psychological distress among Chinese rural population: A moderated mediation model of sleep quality and age. PLoS ONE. 2020;15(10): e241301. 10.1371/journal.pone.0241301 PMC7598484 33125424

[34] Le MTH, Tran TD, Holton S, Nguyen HT, Wolfe R, Fisher J. Reliability, convergent validity and factor structure of the DASS-21 in a sample of Vietnamese adolescents. PLoS ONE. 2017;12(7): e180557. 10.1371/journal.pone.0180557 PMC5516980 28723909

[35] Mishra P, Negi MPS, Srivastava M, Singh K, Rajender S. Decline in seminal quality in Indian men over the last 37 years. Reprod Biol Endocrin. 2018;16(1):103. 10.1186/s12958-018-0425-z PMC6199708 30352581

[36] Sengupta P, Borges EJ, Dutta S, Krajewska-Kulak E. Decline in sperm count in European men during the past 50 years. Hum Exp Toxicol. 2018;37(3):247–55. 28413887 10.1177/0960327117703690

[37] Rosa-Villagran L, Barrera N, Montes J, Riso C, Sapiro R. Decline of semen quality over the last 30 years in Uruguay. Basic Clin Androl. 2021;31(1):8. 33952196 10.1186/s12610-021-00128-6 PMC8101031

[38] Han B, Wang J, Geng Y, Shen L, Wang H, Wang Y, Wang M. Chronic Stress Contributes to Cognitive Dysfunction and Hippocampal Metabolic Abnormalities in APP/PS1 Mice. Cell Physiol Biochem. 2017;41:1766–76. 28365686 10.1159/000471869

[39] DeBerardinis RJ, Chandel NS. We Need to Talk about the Warburg Effect. Nat Metab. 2020;2:127–9. 32694689 10.1038/s42255-020-0172-2

[40] Sanocka D, Kurpisz M. Reactive oxygen species and sperm cells. Reprod Biol Endocrin. 2004;2:12. 10.1186/1477-7827-2-12 PMC400757 15038829

[41] Browning JD, Horton JD. Molecular Mediators of Hepatic Steatosis and Liver Injury. J Clin Invest 2004, 114. 10.1172/JCI22422 PMC449757 15254578

[42] Yuan C, Wang J, Lu W. Regulation of semen quality by fatty acids in diets, extender, and semen. Front Vet Sci. 2023;10:1119153. 37180054 10.3389/fvets.2023.1119153 PMC10174315

[43] Sugden MC, Holness MJ. Recent Advances in Mechanisms Regulating Glucose Oxidation at the Level of the Pyruvate Dehydrogenase Complex by PDKs. Am J Physiol-Endoc M. 2003;284:E855–62. 10.1152/ajpendo.00526.2002 12676647

[44] Grundkeiqbal I, Iqbal K, Tung Yc, Quinlan M, Wisniewski Hm, Binder Li. Abnormal Phosphorylation of the Microtubule-Associated Protein Tau (tau) in Alzheimer Cytoskeletal Pathology. Proceedings of the National Academy of Sciences 1986, 83: 4913–4917. 10.1073/pnas.83.13.4913 PMC323854 3088567

[45] Vellani E, Colasante A, Mamazza L, Minasi MG, Greco E, Bevilacqua A. Association of state and trait anxiety to semen quality of in vitro fertilization patients: a controlled study. Fertil Steril. 2013;99(6):1565–72. 23414918 10.1016/j.fertnstert.2013.01.098

[46] Anderson K, Nisenblat V, Norman R. Lifestyle factors in people seeking infertility treatment - A review. Aust Nz J Obstet Gyn. 2010;50(1):8–20. 10.1111/j.1479-828X.2009.01119.x 20218991

[47] Ragni G, Caccamo A. Negative effect of stress of in vitro fertilization program on quality of semen. Acta Eur Fertil. 1992;23(1):21–3. 1293895

[48] Eskiocak S, Gozen AS, Yapar SB, Tavas F, Kilic AS, Eskiocak M. Glutathione and Free Sulphydryl Content of Seminal Plasma in Healthy Medical Students During and after Exam Stress. Hum Reprod 2005, 20. 10.1093/humrep/dei062 15890736

[49] Odetayo AF, Akhigbe RE, Bassey GE, Hamed MA, Olayaki LA. Impact of Stress on Male Fertility: Role of Gonadotropin Inhibitory Hormone. Front Endocrinol 2024, 14. 10.3389/fendo.2023.1329564 PMC10801237 38260147

[50] Gollenberg AL, Liu F, Brazil C, Drobnis EZ, Guzick D, Overstreet JW, Redmon JB, Sparks A, Wang C, Swan SH. Semen Quality in Fertile Men in Relation to Psychosocial Stress. Fertil Steril. 2010;93:1104–11. 19243749 10.1016/j.fertnstert.2008.12.018

[51] Corona G, Giagulli VA, Maseroli E, Vignozzi L, Aversa A, Zitzmann M, Saad F, Mannucci E, Maggi M. Testosterone Supplementation and Body Composition: Results from a Meta-Analysis of Observational Studies. J Endocrinol Invest 2016, 39. 10.1007/s40618-016-0480-2 27241317

[52] Zhu Z, Li R, Feng C, Liu R, Zheng Y, Hoque SAM, Wu D, Lu H, Zhang T, Zeng W. Exogenous Oleic Acid and Palmitic Acid Improve Boar Sperm Motility via Enhancing Mitochondrial Beta-Oxidation for ATP Generation. ANIMALS-BASEL 2020, 10(4). 10.3390/ani10040591 PMC7222800 32244409

[53] Silva B, Mantha OL, Schor J, Pascual A, Placais P, Pavlowsky A, Preat T. Glia fuel neurons with locally synthesized ketone bodies to sustain memory under starvation. Nat Metab. 2022;4(2):213–24. 35177854 10.1038/s42255-022-00528-6 PMC8885408

[54] Khang AR, Kim DH, Kim M, Oh CJ, Jeon J, Choi SH, Lee I. Reducing Oxidative Stress and Inflammation by Pyruvate Dehydrogenase Kinase 4 Inhibition is Important in Prevention of Renal Ischemia-Reperfusion Injury in Diabetic Mice. Diabetes Metab J 2024, 48. 10.4093/dmj.2023.0196 PMC11140394 38311057

[55] Connaughton S, Chowdhury F, Attia RR, Song S, Zhang Y, Elam MB, Cook GA, Park EA. Regulation of pyruvate dehydrogenase kinase isoform 4 (PDK4) gene expression by glucocorticoids and insulin. Mol cell endocrinol. 2010;315(1–2):159–67. 19703515 10.1016/j.mce.2009.08.011 PMC2815206

[56] Odetayo AF, Akhigbe RE, Bassey GE, Hamed MA, Olayaki LA. Impact of stress on male fertility: role of gonadotropin inhibitory hormone. Front endocrinol. 2023;14:1329564. 10.3389/fendo.2023.1329564 PMC10801237 38260147

[57] Visconti PE. Sperm Bioenergetics in a Nutshell1. Biol Reprod 2012, 87. 10.1095/biolreprod.112.104109 PMC3464909 22914312

[58] Luo X, Guo Y, Li X, Mei Z, Zhou H, Qiu P, Wang H, Chen Y, Gong Y. Aromatase Reduces Sperm Motility by Down-Regulating the Expression of Proteins Related to ATP Synthesis in Seminal Plasma Extracellular Vesicles. BMC Genomics. 2025;26:1–13. 40155807 10.1186/s12864-025-11500-5 PMC11951553

